# Differential diagnosis of bulbous nasal tip – gouty tophus: a case report

**DOI:** 10.1016/j.bjorl.2026.101879

**Published:** 2026-08-12

**Authors:** Barbara Guidolin Froner, Caroline Cardoso Gusson Figueiredo, Marcos Alexandre Matsumoto Gallo, Beatriz Ferreira Maraccini

**Affiliations:** Instituto Penido Burnier, Campinas, SP, Brazil

## Introduction

The nose is composed of skin, bone, cartilaginous framework, and mucosa; its topography is defined by convexities, concavities, and depressions that reflect the underlying skeletal structure. All changes related to the position of the nasal tip can be anticipated by considering the tip as a tripod, which may influence shape, rotation, projection, symmetry, and positioning.^1^

Several conditions can compromise nasal tip definition, including nasal granulomatosis, congenital malformations, benign midline tumors, and gouty tophi. Gout is the most common microcrystal-induced arthropathy, primarily affecting men between 30- and 50-years of age, and reflects abnormal uric acid metabolism.^2^

The diagnosis is established in the presence of hyperuricemia, gouty tophi, acute arthritis attacks, or chronic arthritis, but it may be clinically challenging, particularly when occurring in atypical locations. The gold standard for diagnosis is the identification of needle-shaped, negatively birefringent monosodium urate crystals in tissue or synovial fluid using polarized light microscopy.^3^

Conventional Computed Tomography (CT) is sensitive for detecting bone erosions, whereas Magnetic Resonance Imaging (MRI) typically shows an intermediate-signal mass, depending on calcium concentration. However, these findings are not specific for gout.^4^

## Case report

A 57-year-old white male from Paulínia, Brazil, employed as a private driver, presented with progressive enlargement of the nasal tip and a palpable, painless lesion in the left nasal cavity. The lesion had been slowly evolving over 7-years, without associated obstruction, discharge, crusting, or epistaxis. He denied other lesions, nasal trauma, or illicit drug use. Past history included septoplasty and bilateral inferior turbinectomy 20-years earlier, and surgical excision of an elbow tophus.

On physical examination, the nasal dorsum was straight in its bony portion and convex in the cartilaginous portion. The tip appeared bulbous, ill-defined, and asymmetric, with thick skin and asymmetric nostrils. Anterior rhinoscopy revealed a smooth-surfaced, whitish, non-pulsatile lesion measuring approximately 1 cm, protruding into the left nasal vestibule and partially into the right, confined to soft tissues.

Laboratory evaluation revealed mild anemia (hemoglobin 11.2 g/dL), elevated Erythrocyte Sedimentation Rate (ESR) (84 mm/h; upper limit 15 mm/h), negative rheumatological tests (ANA, p-ANCA, and c-ANCA), and negative serological tests for syphilis and leishmaniasis.

**Fig. 1:**
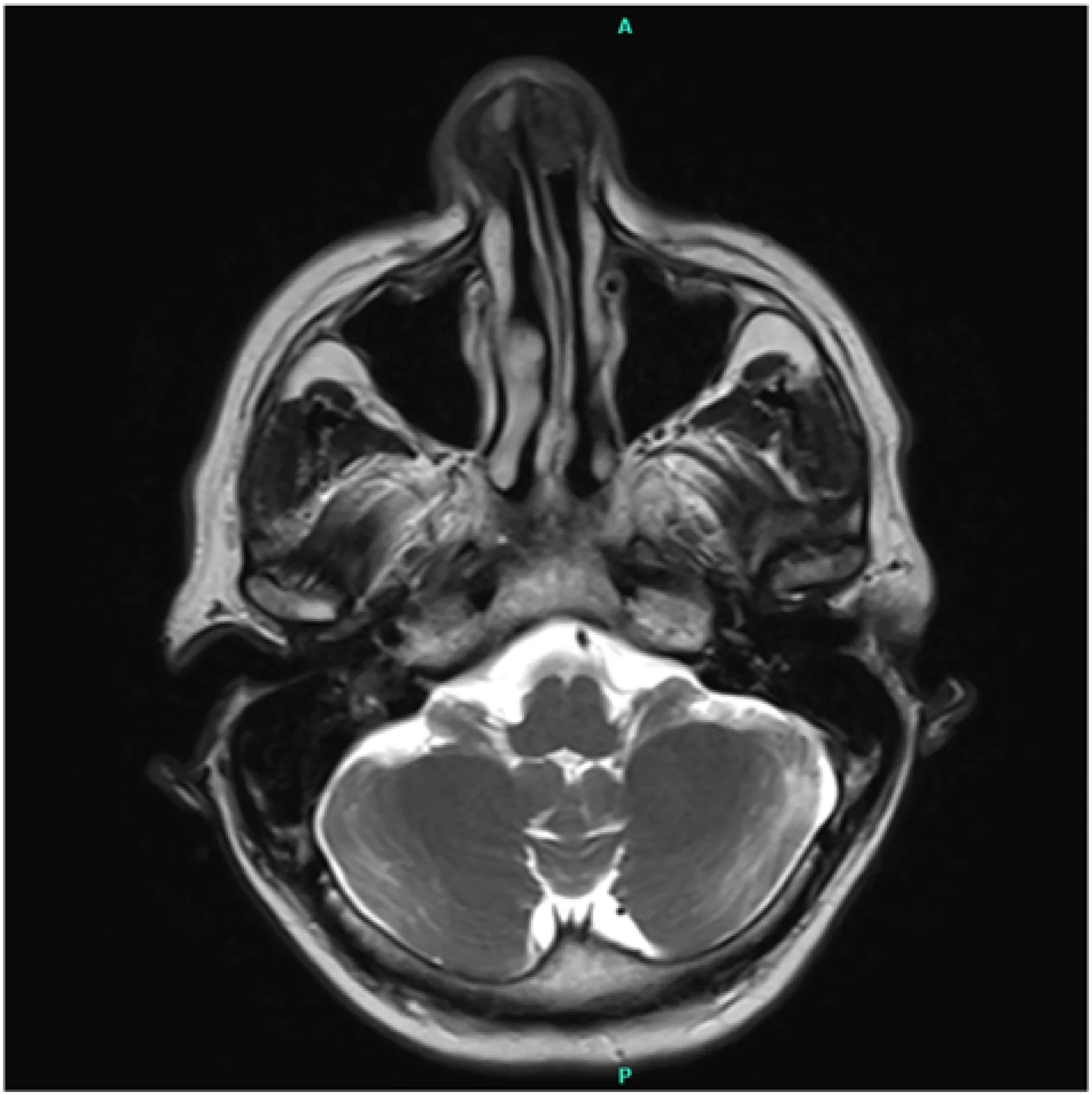
Axial T2-weighted MRI demonstrating a heterogeneous anterior nasal cavity lesion.

**Fig. 2:**
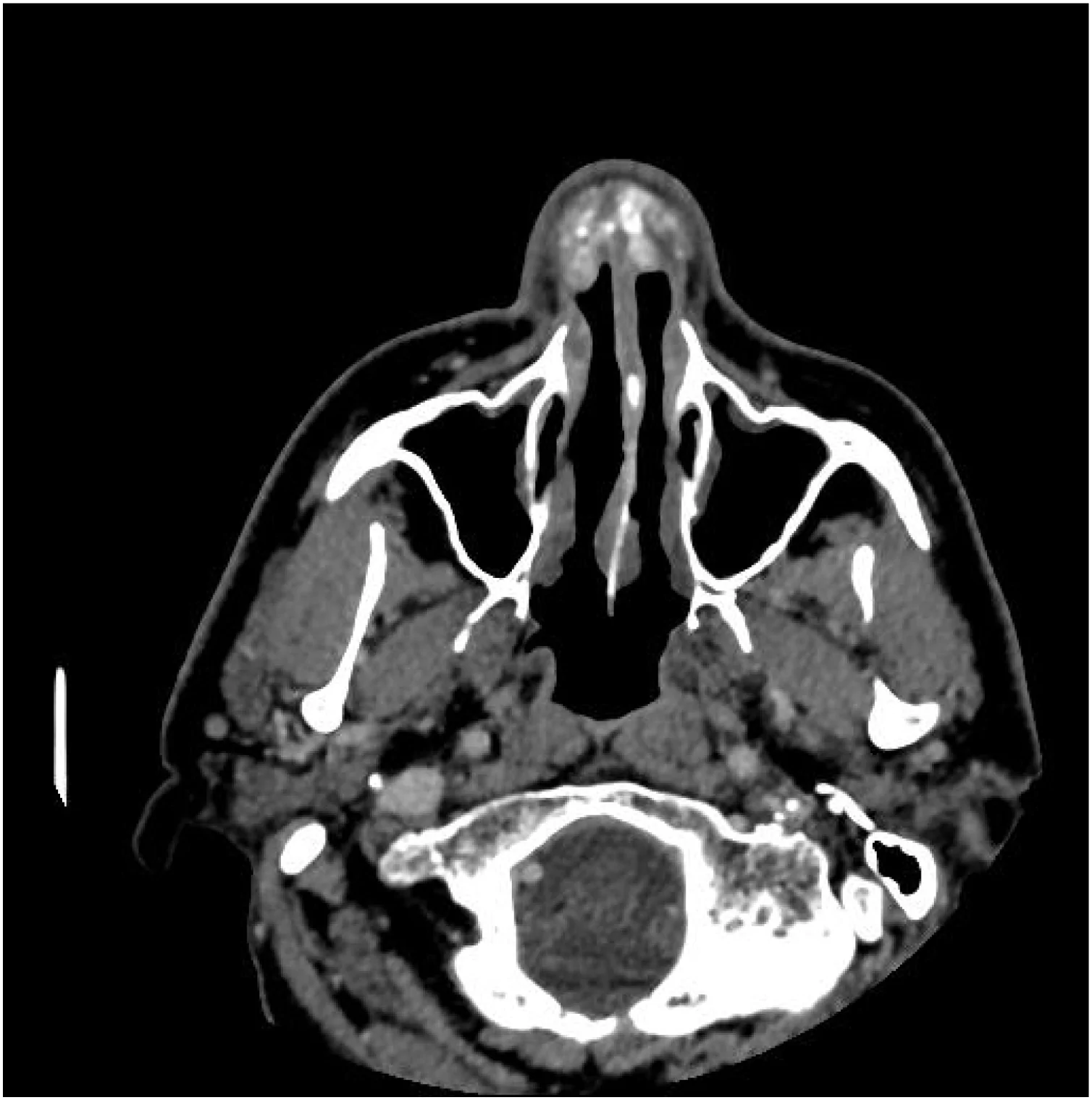
Axial section showing lesion with soft tissue density and areas of calcification.

MRI of the face revealed a heterogeneous lesion in the anterior nasal cavity associated with thickening of the overlying skin and subcutaneous tissue of the nasal dorsum and tip (Fig. 1). Paranasal sinus CT demonstrated a lobulated, solid soft tissue–density lesion with areas of calcification, without contrast enhancement, occupying the superior portion of both nasal cavities (predominantly on the left), measuring 2.0 × 1.5 cm (Fig. 2).

Incisional biopsy revealed a chronic granulomatous inflammatory process with amorphous material, consistent with a foreign body-type reaction. Fungal and mycobacterial cultures were negative. Histology showed chronic lymphoplasmacytic-histiocytic infiltrate with multinucleated giant cells and epithelioid macrophages.

**Fig. 3:**
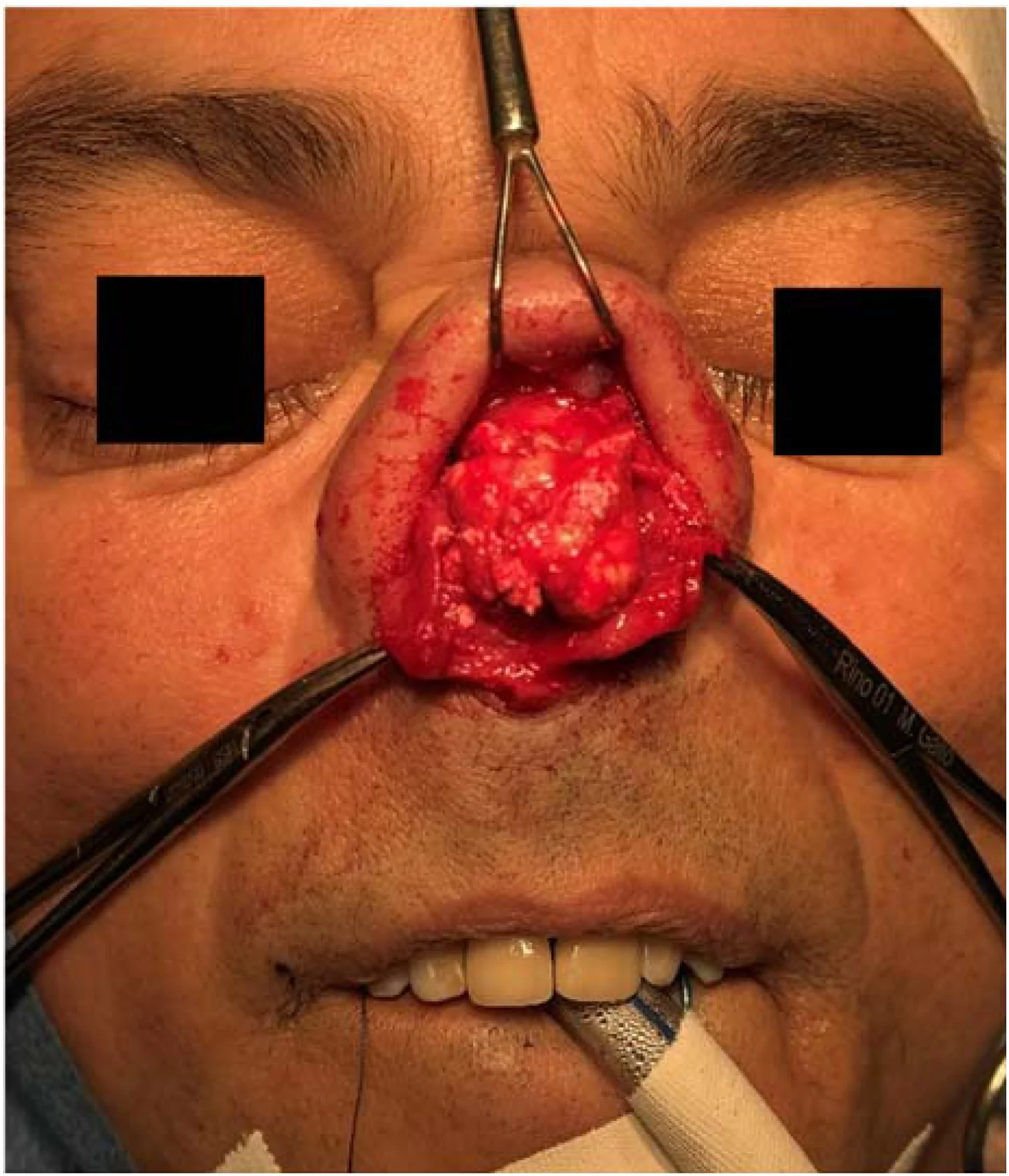
Open rhinoplasty.

**Fig. 4:**
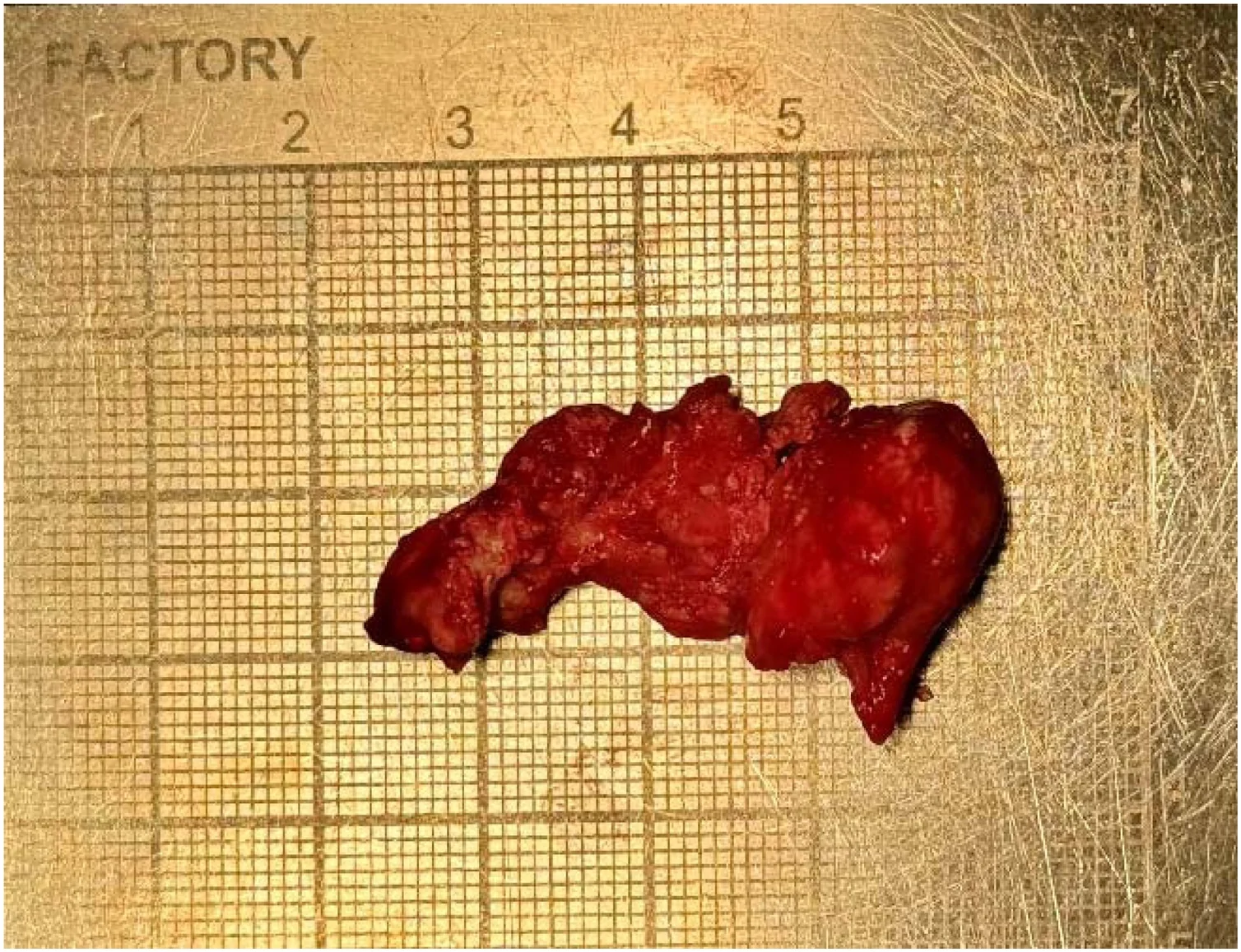
The specimen measured approximately 4 × 2 cm.

The lesion was excised through open rhinoplasty. It was located at the cartilaginous dorsum and involved the upper and lower lateral cartilages, with preservation of the bony dorsum (Fig. 3). The specimen measured approximately 4 × 2 cm (Fig. 4), grossly appearing as a white, encapsulated, soft mass, and was submitted for histopathological evaluation. Bilateral spreader grafts, tongue-in-groove maneuver, interdomal suture, and alar-spanning suture were performed for nasal tip refinement.

The patient was followed up postoperatively with satisfactory functional and aesthetic outcomes.

## Discussion

The surgical approach in rhinoplasty refers to the method used to expose the nasal tip structures, allowing direct visualization, assessment, and modification.^1^

Gout most commonly affects the first Metatarsophalangeal (MTP) joint, whereas involvement of the head and neck region is rare. In such uncommon presentations, the differential diagnosis includes skin neoplasms and chondroid lesions, requiring histopathological confirmation.^5^

The development of gouty tophi reflects chronic failure in serum uric acid control; therefore, postoperative risk factor modification is essential to achieve favorable long-term outcomes.^3^

Management of gouty tophi includes lifestyle modifications and pharmacological therapy. However, when medical treatment or patient adherence is inadequate, surgical excision is indicated.^2^

Successful nasal tip surgery requires a thorough understanding of the lower lateral cartilage anatomy, its relationship with adjacent soft tissues, nasal tip support mechanisms, and the effects of each surgical maneuver. The open approach provides optimal exposure of structural deformities, thereby facilitating surgical correction.^1^

In the present case, after tumor excision, extensive deformity of the nasal tip architecture was observed due to cartilaginous resorption. Surgery was indicated both for tumor removal with histopathological analysis and for restoration of nasal aesthetics. The patient was informed and provided consent regarding potential aesthetic changes related to the procedure.

**Fig. 5:**
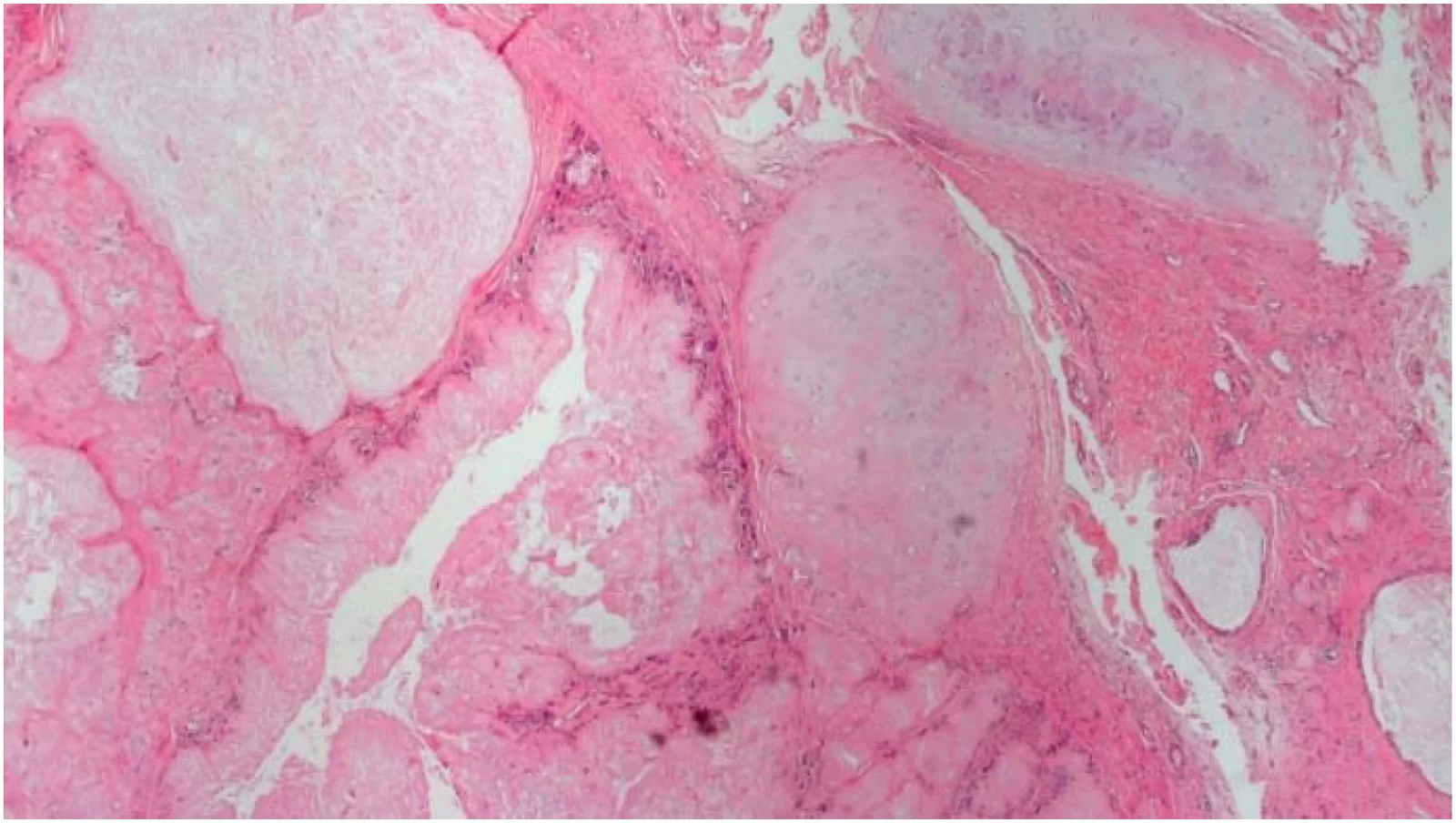
Histopathological examination showing nodular granulomatous aggregates of acellular, amorphous eosinophilic material surrounded by palisading histiocytes and multinucleated giant cells.

Histopathological analysis revealed nodular aggregates with a granulomatous appearance, composed of acellular, amorphous, pale eosinophilic material surrounded by a palisading arrangement of histiocytes and multinucleated giant cells (Fig. 5). Additionally, a feathery appearance in some deposits due to needle-shaped empty spaces, combined with the patient’s history of gout, allowed the diagnosis of nasal gouty tophus.

## Conclusion

Gouty tophus should be considered in the differential diagnosis of nasal tip lesions. Surgical excision with histopathological analysis is essential for definitive diagnosis and management.

## ORCID ID

Marcos Alexandre Matsumoto Gallo: 0009-0009-2398-0682

Beatriz Ferreira Maraccini: 0009-0005-0419-9111

## Funding

This research received no external funding and was self-financed by the authors.

## Data availability

The authors declare that all data are available in repository.

## Declaration of competing interest

The authors declare no conflicts of interest.
